# Agronomic Assessment of a Controlled-Release Polymer-Coated Urea-Based Fertilizer in Maize

**DOI:** 10.3390/plants10030594

**Published:** 2021-03-22

**Authors:** Ricardo Gil-Ortiz, Miguel Ángel Naranjo, Antonio Ruiz-Navarro, Marcos Caballero-Molada, Sergio Atares, Carlos García, Oscar Vicente

**Affiliations:** 1 Institute for Plant Molecular and Cell Biology (UPV-CSIC), Universitat Politècnica de València, The Spanish National Research Council, 46022 Valencia, Spain; mnaranjo@ibmcp.upv.es (M.Á.N.); marcamo2@ibmcp.upv.es (M.C.-M.); 2 Fertinagro Biotech S.L., Polígono de la Paz, C/Berlín s/n, 44195 Teruel, Spain; sergio.atares@tervalis.com; 3 Centre for Soil and Applied Biology Science of Segura, The Spanish National Research Council, (CEBAS-CSIC), Espinardo University Campus, 30100 Murcia, Spain; ruiznavarro@cebas.csic.es (A.R.-N.); cgarizq@cebas.csic.es (C.G.); 4 Institute for the Conservation and Improvement of Valencian Agrodiversity (COMAV), Universitat Politècnica de València, 46022 Valencia, Spain; ovicente@upvnet.upv.es

**Keywords:** coated-urea fertilizer, humic acid, lignosulfonate, natural polymers, seaweed extract, maize

## Abstract

Increasing nutrient use efficiency of fertilizers is one of the major challenges to improve crop yields and minimize environmental impacts. This work compared the efficacy of a new ecological polymer-coated urea fertilizer and a slow release urea-based traditional fertilizer. Reductions in the N doses of the polymer-coated fertilizer were tested. A comparative study was first carried out by measuring the different physiological and yield parameters at the micro-scale level, and later-on field experiments were performed. Grain yield in the field was significantly higher (20%) when applying the new controlled-release fertilizer than when using the traditional one at the same dose. A 20% reduction in N content in the new fertilizer gave similar physiological and yield responses compared to the traditional fertilizer. We conclude that this new fertilizer can be used in extensive cropping of maize, guaranteeing at least the same yields than traditional fertilizers, with a reduction on the impact on soil properties and nitrogen losses.

## 1. Introduction

Maize (*Zea mays* L.) is the most widely grown cereal in the world in terms of production and yield, followed by wheat and rice [1]. According to FAO, maize grain production has increased by 25% since 2010. Production was around 1.1 billion tons in 2019, and this yield is expected to be maintained, and even increase, in the forthcoming years. Other uses of maize include making feed, and it is used as green fodder, silage or for other industrial uses [2]. However, the increasing demand of cereals and reduced cultivated surfaces render it necessary to increase the crop yields per unit area [3,4]. In cereals, fertilizer research has focused mainly on increasing nutrient use efficiency (NUE) in recent decades [5,6,7,8]. The necessary successive top-dressing applications during cultivation are usually based on the fact that most so-called traditional fertilizers are not very stable and have low constant N-release kinetics for plant development [9,10]. High losses tend to occur at the beginning of N fertilizer applications through volatilization, denitrification, or leaching [5]. Maize yield is highly affected by crop agronomic management, and water use efficiency and fertilization are the most important variables [11,12]. In fact, one of the most relevant environmental problems in maize cultivation is caused by eutrophication polluting aquifers [13]. N fertilization requirements are high and close to 300 kg of N per ha to produce about 10 tons of maize grain. Slow release (SRF) or controlled-release (CRFs) fertilizers have been used to increase the efficient use of nutrients by crops and to reduce losses, especially N losses due to its high mobility in soil [14,15,16,17]. CRFs, unlike SRFs, are not so dependent on soil microbiology and are more efficient in providing nutrients to the plants [18]. The idea of manufacturing CRFs is based on improving crop nutritional status by applying only the amount of fertilizer needed for crop development. Different synthetic CRFs have been developed in the past, such as urea formaldehyde, isobutylidenediurea (IBDU), crotonylidenediurea, or sulfur-coated urea [19]. A single CRF application during crop establishment is usually enough to cover its biological requirements and cut management costs [20]. In recent years, this technology has been successfully used in high-value crops like horticulture, ornamental, or wood production [21,22,23]. However, the high manufacturing costs and low degradability of synthetic-based materials make their application in cereals unfeasible. This is why the use of waste products produced in the wood industry as natural resins has been considered an alternative to synthetics. In fact, eco-friendly fertilizers like those based on starch and cellulose, and their derivatives, or on lignin or agricultural residues, have been successfully used to slow down N release from urea [24,25,26,27]. Urease inhibitors mixed in urea-based formulations successfully diminish urea hydrolysis and prolong fertilizer life [28,29,30,31,32]. The use of biostimulants as amino acids, humic and/or fulvic acids, or algae extracts increases the resistance of crops against abiotic stress [33,34,35].

The objective of this research is to compare the efficacy of a new ecological controlled-release, coated urea fertilizer with traditional ones applied to maize, in physiological and grain yield/quality terms. The novelty of this research lies in combining the same fertilizer made of eco-friendly polymers and byproducts from wood pulp production with a urease inhibitor and natural biostimulants.

## 2. Results

### 2.1. Plant Growth, Leaf Greenness, and Effective Quantum Yield of Photosystem II

For the microscale experiment, the results of the growth parameters, ΦPSII, leaf greenness, and N content between the different applied fertilizer treatments are shown in Table 1. In the stage of eight nodes, there were no significant differences in the studied growth parameters between CRF, DURAMON^®^ and ammonium nitrosulfate (NSA). CRF reductions showed variable results that did not correlate with the applied N quantity, and no significant differences appeared between them. However, a significant reduction took place in the primary stem length for CRF_r2_ and the total foliar area for CRF_r1_, CRF_r2_, and CRF_r4_ compared to the CRF applied at the maximum dose. Thus, no significant correlation was observed between ΦPSII and leaf greenness. Foliar N content measured by the N-pen non-destructive technique was 1.2-fold significantly higher for treatments CRF and NSA compared to DURAMON^®^.

### 2.2. Foliar Nutrient Content

**Figure 1:**
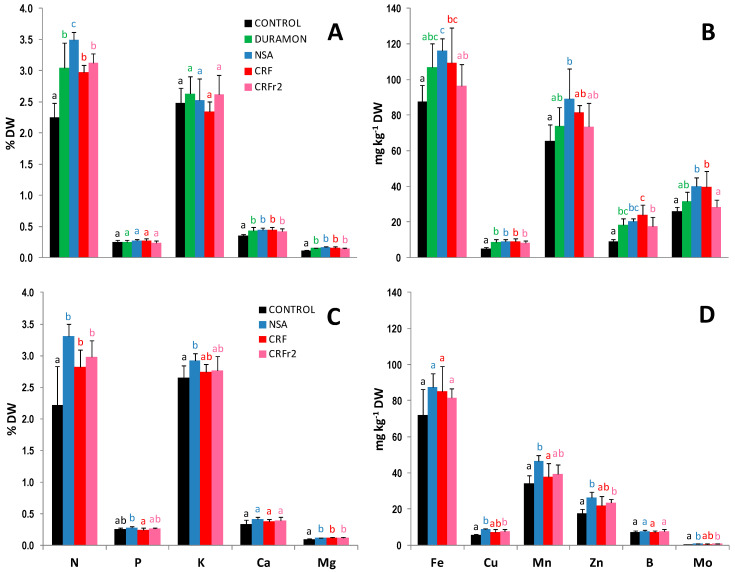
Macro- and micronutrient foliar content comparisons of the maize fertilized with CRF, CRF_r2_, DURAMON^®^, ammonium nitrosulfate (NSA) and the untreated CONTROL on the microscale (**A**,**B**) and the field scale (**C**,**D**) experiments. The results for macronutrients (N, P, K, Ca, and Mg) were expressed as % leaf dry weight (DW) and micronutrients (Fe, Cu, Mn, Zn, and B) as mg kg^−1^ leaf DW (Microscale- CRF, DURAMON^®^ and NSA: 350 kg ha^−1^; CRF_r2_: 280 kg ha^−1^. Field- CRF, NSA: 300 kg ha^−1^; CRF_r2_: 240 kg ha^−1^). Values are means ± SD (*n* leaf pools per treatment = 4). Different letters for a specific macro- or micronutrient in each panel indicate statistically significant differences between treatments (ANOVA, *p* < 0.05).

The results obtained for the macro- and micronutrient foliar contents on the microscale and field-scale experiments, in the stage of eight nodes are shown in Figure 1. N content on the microscale came close to being 1.2-fold significantly higher in the plants treated with NSA, as compared to CRF, CRF_r2_, and DURAMON^®^. For P, K, Ca, and Mg, no significant differences between the other applied treatments were found. Although the foliar N content in the NSA-treated plants was slightly higher on average, compared to CRF and CRF_r2_, no significant differences appeared. Micronutrient content was significantly reduced in the plants treated with CRF_r2_ compared to CRF for Fe, Zn, and B on the microscale. In the field, no significant differences appeared in the micronutrient content between the applied fertilizer treatments.

### 2.3. Hormone Activity

The hormone activity comparison made of the applied fertilizer treatments is shown in Table 2. On the microscale, the *IAA* foliar content came close to being 2-fold higher for treatments CRF and DURAMON^®^, compared to NSA. *JA* content in the presence of DURAMON^®^ was 2.2-, 3.1-, and 2.6-fold higher than with CRF, CRF_r2_, and NSA, respectively. Conversely, 2.4- and 3.6-fold significantly lower *tZ* levels were detected in the DURAMON^®^-treated plants compared to CRF and NSA. *CK* content for CRF_r2_ was 3.7- and 4.7-fold significantly lower in leaves for *iP* and *tZ* vs. CRF. In the field, significant 1.6-, 1.9- and 4-fold increases took place in the content of *iP*, *DHZ*, and *JA* for the CRF-treated plants compared to those of NSA. The *GA_3_* levels were 3.2-fold significantly higher in the treatments applied with NSA vs. CRF.

### 2.4. Growth, Yield, and Cereal Grain Composition

**Figure 2:**
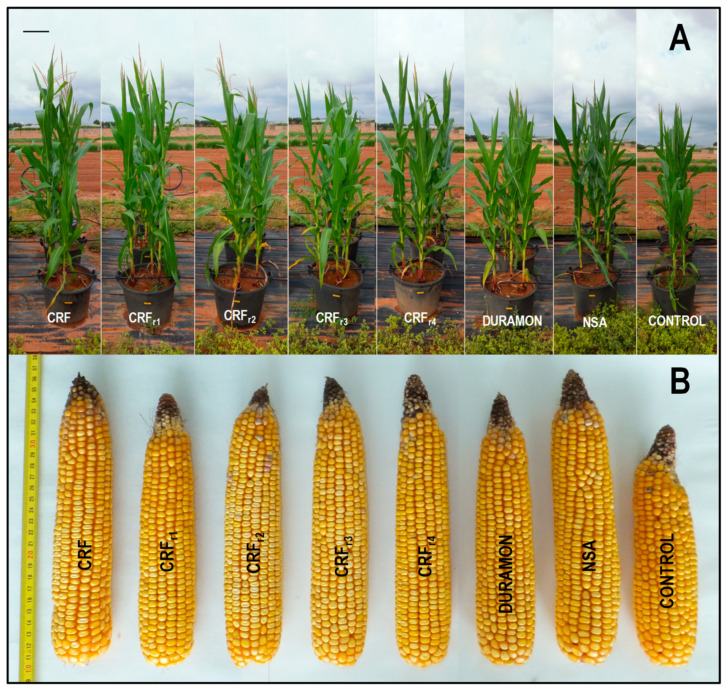
Maize responses in the growth (**A**) and ear size (**B**) to the applied fertilizers (CRF, CRF_r1_-CRF_r4_, DURAMON^®^ and NSA) compared to CONTROL at the microscale (CRF, DURAMON^®^ and NSA: 350 kg ha^−1^; CRF_r1_: 315 kg ha^−1^; CRF_r2_: 280 kg ha^−1^; CRF_r3_: 245 kg ha^−1^; CRF_r4_: 210 kg ha^−1^). Metrics in figure (**B**) correspond to cm.

On the microscale, no significant differences appeared in the grain yield produced for the CRF, DURAMON^®^ and NSA applied fertilizer treatments (Figure 2, Table 3). However, the dry weight of the aerial part was 1.1-fold significantly higher in the DURAMON^®^-treated plants than in NSA. The ear weight for DURAMON^®^ was 1.2-fold significantly lower, but in the field (Table 4), ear and grain dry weights were 1.3- and 1.2-fold higher in the CRF plants vs. NSA. No significant differences were observed for any studied parameter between the CRF_r2_- and NSA-treated plants, or for grain composition between the treatments and the CONTROL. As a result, on the microscale, the values of the measured parameters for CRF were 1.3% ash, 14.1% humidity, 1.8% lipids, 6.9% protein, 2.4% crude fiber, and 73.5% total carbohydrates on average.

## 3. Discussion

In developing new highly efficient, enviro-friendly and low-cost fertilizers, attempts have been made to enhance NUE and minimize the environmental contamination by applying SRFs or CRFs to crops with a high added value, such as horticultural, ornamental or for wood [36,37,38,39]. This allows the slow release of nitrogen, whose emission kinetics fall more in line with the crop’s nutritional requirements [14,40]. In maize, based on the bibliography, different coated fertilizers have been proved successful in controlling N release, improving physiological activities, and consequently increasing yields [41,42,43,44]; for example, better physiological results were obtained using CRFs plus urease inhibitors compared to traditional fertilizers [45,46,47]. Currently, most fertilizers in the market are based on synthetic materials, contrary to that analyzed in the present study, a CRF coated with a mix of lignosulfonates—natural resins obtained as by-products from wood industries—and humic acids. Related to our study, some research was done with fertilizers based on raw materials like starch, lignin, or agricultural residues, increasing NUE [48,49]. The main objective of this study was improving the controlled release of N to increase the yields and reduce the contamination, by an improvement in the NUE, as one of the main objectives when setting up fertilization programs [50,51,52]. First, the applied fertilizers (NSA, DURAMON^®^ and the CRF) were compared for their effectiveness on the microscale, to determine their effects and also which is the best N-dose reduction in the CRF, and later the experiments were field-scaled. On the microscale experiment, no significant differences were observed in the physiological stage of eight nodes when comparing the different growth parameters between the three fertilizers (Table 1). With respect to the photosynthetic parameters quantified by using non-destructive techniques (ΦPSII and leaf greenness), no significant differences were found between the different treatments at the higher concentrations. N foliar content came close to being 18% significantly higher in the plants treated with NSA and CRF vs. DURAMON^®^. Reductions in CRF showed a slight reduction in the N content, only significant with CRF_r1_ and CRF_r3_, but not showing a clear pattern. Differences in N-foliar content before flowering, quantified by the technique ICP-OES (Figure 1), showed levels into sufficiency, between 2.75 and 3.25% [53]. The highest significant N levels were obtained with the NSA treatment, being between ca. 10 and 15% higher compared to CRF and CRF_r2_ treatments, in both, at microscale and field; and, also, with respect to DURAMON^®^ at the microscale. This high efficiency of NSA is justified by the fast N-NO_3_^−^ release, but its poor N-release kinetics makes compulsory additional applications at top-dressing to guarantee yields. No significant differences in the rest of macro- and micronutrient content in leaves were observed between the applied fertilizers (Figure 1). This can be explained because the applied fertilizers were only N-based, and the chelating properties of humic acids, only applied during one season, did not suffice to increase nutrient efficiency with the poor soils used in the experiments. The application of humic acids, highly present in organic products used as amendments or fertilizers, is widely used to improve plant growth, water and nutrient retention in the soil based on their chelating properties [54,55]. Concerning the hormone content (Table 2), *IAA* significantly increased ca. 50% in the CRF- and DURAMON^®^-treated plants compared to NSA, which favored the development of lateral and adventitious roots. The most relevant finding in hormone content was the increase in *CKs* in the CRF-treated plants. *CKs* promote cell division and differentiation, which are fundamental for regulating various physiological processes, such as photosynthesis, growth regulation, or resistance to pathogens [56]. These effects are synergistic with the addition of humic substances that may promote plant development by stimulating root and shoot growth [57,58]. In the field, significant increases of 60%, 90%, and 400% took place in the contents of *iP*, *DHZ*, and *JA*, respectively, for the CRF-treated plants as compared to NSA.

With respect to yields, maize has the best chance of achieving high yields because of its excellent capacity to produce more dry matter per hectare than the rest of cereals. This is because maize is a C_4_ plant with a high photosynthesis rate, 50–60 mg CO_2_/dm^2^ leaf x hour under optimal light intensity and high-temperature conditions (≥30 °C) [59]. Maize is very water-demanding, which is its main limiting factor [60]. However, with non-limited irrigation, yields are conditioned mainly by fertilization, with average ranges between 3 t grain ha^−1^ on dry land and 8 t ha^−1^ on irrigated land, although 12–15 t grain ha^−1^ can be achieved in good areas. In cereal fertilization, particularly in maize, N is the most important element to increase yield [2]. The amount of nitrogenous fertilizer to be applied depends on crop extractions (2.5 kg N Qm^−1^ grain) and expected yields. In practice, up to 350 kg N ha^−1^ is applied to obtain maximum yields. Our results, on the microscale (Table 3), revealed no significant differences in the grain yields among the applied CRF, DURAMON^®^, and NSA fertilizer treatments. The most notable finding was that with 20% less N in the CRF_r2_ treatment, the same quantity of grain was achieved compared to NSA and DURAMON^®^. This is environmentally important to minimize contamination and, accordingly, many different materials have been employed to manufacture less polluting enviro-friendly fertilizers [27,61]. In the field (Table 4), the results on the obtained yield were quantitatively different from those obtained at the microscale. These differences could be explained by the different growing conditions in the two experiments: how the plants were grown (in pots at the microscale/directly in the soil in the field), soil composition, climate, irrigation, and maximum doses applied. Anyway, both experiments reflected proportionally the same differences in the efficacy of the studied fertilizers. CRF had a significantly increased grain dry weight of 17.5% and 16% higher than NSA and CRF_r2_. This means that controlling N release and increasing N dose the fertilizer can be made more long-lasting. In practice, it was confirmed that reducing the N-dose in CRF by 20% it is possible to obtain the same yields that with NSA at the standard dose. The total produced grain came close to the theoretically expected amount for the CRF treatment. According to these results, NUE was increased by 20% with respect to NSA, applying CRF_r2_. The benefits of only one basal application and the reduction of N-dose that consequently reduce contamination, make this new fertilizer highly promising to be applied in extensive maize cultivation.

## 4. Materials and Methods

### 4.1. Plant Material and Experimental Design

#### 4.1.1. Microscale Experiment

To study the effectiveness of the different fertilizers, *Zea mays* var. *indentata* was grown at the microscale under environmental conditions, from spring to fall 2015 in the facilities of the Valencian Institute of Agrarian Research (Moncada, Valencia, Spain). Sowing was carried out in four pots per treatment of dimensions 48 cm—high × 53 cm—Ø and a frame of 60 × 20 cm. They were placed at random on 25 May. Four seeds per pot were grown at a distance of 20 cm and were irrigated with distilled water.

#### 4.1.2. Field Experiment

*Zea mays* var. *Pioneer p0725 short cycle 450*, adapted to the growing conditions in the area, was grown in three blocks of 100 m^2^ in an experimental plot located in Teruel, Spain (GPS coordinates 40°09′41.1″ N 0°45′48.2″ W). Four replications per fertilizer and the CONTROL were established at random for each block on surfaces of 24 m^2^ in a 4 × 4 grid. Sowing was performed in a frame measuring 70 × 12 cm on 3 June 2016 and sprinkler irrigation was applied using well water.

### 4.2. Applied Fertilizers and Treatments

Different nitrogen fertilizers, developed by Fertinagro Biotech S.L. (Teruel, Spain) were tested to compare their efficacy: (i) a SRF, DURAMON^®^ (24% nitrogen—0% phosphorous—0% potassium), composed of urea, including a urease inhibitor (monocarbamida dihidrogenosulfate—MCDHS) with no coating (ES 2 204 307 Spanish patent/WO 2007/132,032 A1 international patent). MCDHS inhibits the transformation of N-urea into NH_4_^+^-N, reducing losses. Also, NH_4_^+^-N is protected, reducing its volatilization and loss, by pH control owing to the microacidification produced by H^+^ release during hydrolysis of the MCDHS molecule; (ii) a controlled-release fertilizer (CRF, hereafter) (24-0-0), based on DURAMON^®^ technology—the same used in SRF, based on the urease inhibitor MCDHS –, but also coated with a mix of lignosulfonates and humic acids in the proportion of 3%; and, (iii) a traditional N fertilizer, commonly used in the cultivation area—ammonium nitrosulphate (NSA) (26-0-0). Each grid had the same untreated areas that were taken as CONTROL.

#### 4.2.1. Microscale Experiments

Fertilizers were applied at a maximum dose of 350 kg ha^−1^. CRF and DURAMON^®^ were applied at basal dressing and NSA was fractioned, 40% when plants had stem lengths of 10–20 cm and 60% when they had reached 40–80 cm. In addition, 10% N dose reductions were applied as different treatments until N content dropped to 60% of the maximum doses per experiment (CRF_r1_: 315 kg ha^−1^; CRF_r2_: 280 kg ha^−1^; CRF_r3_: 245 kg ha^−1^; CRF_r4_: 210 kg ha^−1^).

#### 4.2.2. Field Experiments

Fertilizers were applied at a maximum dose of 300 kg ha^−1^ and fractioned in the same way as in the microscale experiments. Doses for each culture were based on those recommended for the cultivated area according to historical yields. CRF was applied at doses of 100% and 80% (CRF_r2_: 240 kg ha^−1^), compared with NSA, which was used as the traditional fertilizer.

### 4.3. Soil Characterization

Several soil properties were measured to characterize the soil used in both experiments. pH and EC were determined in a 1/5 (*w/v*) aqueous soil extract by shaking for two h, followed by centrifugation at 26,916 *g* for 15 min and filtration. pH was measured by a pH meter (Crison mod. 2001, Barcelona, Spain) and EC with a Conductivity meter (Crison micro CM2200, Barcelona, Spain). Total and organic soil C (SOC) and total N (N) were determined by combustion gas chromatography in a Flash EA 1112 Thermo Finnigan (Franklin, MA, USA) elemental analyzer after eliminating carbonate by acid digestion with HCl. The total nutrient contents (P, K, Ca, Mg, Cu, Fe, K, Mg, Mn, and Zn) were extracted by aqua regia digestion (3:1, *v/v*, HCl/HNO_3_) and determined by ICP-AES (Inductively Coupled Plasma Atomic Emission Spectroscopy) (Thermo Elemental Iris Intrepid II XDL, Franklin, MA, USA). The analysis showed that both cultures grew on N-poor soils (Table 5). According to the World Reference Base for Soil Resources [62], these soils, as having an intensive agricultural use, can be classified as Antrosols.

### 4.4. Growth and Photosynthetic Parameters

Differences in maize growth between fertilizer treatments for the microscale experiments were compared in the stage of eight nodes (before flowering) and at the end of culture. The growth parameters studied in the vegetative stage were total fresh weight of the aerial part (g), total length (cm), primary stem length (cm), stem diameter (cm) (measured by a StandardGage calliper—PCE instruments, Spain), leaf weight (g) and total foliar area (cm^2^) (using an LI-3100C area meter—LI-COR^®^, Nebraska, USA). Some plant material was weighed before being dried at 65 °C until a constant mass was obtained to calculate the dry weight percentage. The relative water content was calculated as RWC (%) = (FW − DW)/(TW − DW) × 100, where FW is fresh mass, TW is turgid mass after saturating leaves with water at 4 °C in the dark, and DW is dry mass after oven-drying leaves at 65 °C for 72 h [63]. At harvest, the studied growth parameters were the total fresh weight and dry weight of the aerial part (g), primary stem length (cm) and stem diameter (mm). Leaf greenness was measured by a SPAD-502 Chlorophyll meter (Konica-Minolta, Osaka, Japan) [64] and the effective quantum yield of photosystem II electron transport (ΦPSII) was established by a leaf fluorometer (Fluorpen FP100, Photos System Instrument, Drásov, Czech Republic). The photosynthetic parameters were evaluated in a minimum of 25 leaves per treatment.

### 4.5. Foliar Nutrient Analysis

Foliar analyses were performed with the fresh samples collected on 7 and 20 July for the microscale and the field experiments, respectively. Sampling was carried out at the same time as growth parameters were characterized. Samples comprised the middle 1/3 of fully developed leaves just below the apex. They were cut from one plant per pot on the microscale and from the different plants growing on 1 m^2^ in the field. Four pool-replicates per treatment and culture were collected and kept at −20 °C until biochemical analyses were performed. Compositions in terms of macro- (N, P, K, Ca, and Mg) and micronutrients (Fe, Cu, Mn, Zn, B, and Mo) were determined by inductively coupled plasma optical emission spectrometry (ICP-OES) by the company Eurofins Agroambiental, S.A. (Lleida, Spain), using their own protocols. N content was estimated by an N-Pen N 100 apparatus (Photon System Instruments, Drásov, Czech Republic).

### 4.6. Hormone Activity

The samples used for the foliar nutrient analyses were also employed for determining the activity of the different enzymes related with plant development, such as indoleacetic acid (*IAA*), jasmonic acid (*JA*), salicylic acid (*SA*), abscisic acid (*ABA*), and cytokinins (*CK*), including isopentenyl adenine (*iP*), t-zeatin (*tZ*), and dihidrozeatin (*DHZ*). Analyses were done by the Plant Hormone Quantifying Service (IBMCP-UPV) in a Thermo Scientific™ Q Exactive™ Hybrid Quadrupole-Orbitrap Mass Spectrometer (LC-MS/MS HR), using the Service’s in-house protocols. Hormone content was expressed as ng g^−1^ of leaf dry weight.

### 4.7. Yield and Cereal Grain Composition

After grain ripening was complete on the micro- (10-Sept) and field (21-Sept) scales, the remaining plants per culture were harvested, and grain yield was determined. The parameters evaluated on the microscale, in a total of 12 plants per treatment, were ear weight (g), ear length (cm), total dry grain weight per plant (g), 100-grain weight (g), and grain number per plant/100. For the field experiment, 20 plants were sampled per grid and replication, with a total of 80 plants per treatment; the parameters measured were ear length (cm), ear fresh/dry weight (t ha^−1^), grain dry weight (t ha^−1^). Nitrogen use efficiency (NUE) was calculated as the quotient between the grain yield of the fertilized area, and the quantity of N applied as N fertilizer. Different quality parameters were measured in the grain for each treatment, based on food quality analysis methods (Commission Regulation EC N° 152/2009 of January 27): humidity (gravimetric by drying in an oven at 130 °C), ashes (gravimetric by incineration at 550 °C), lipids (extraction without hydrolysis in Soxtec Avanti—Foss), protein (Kjeldahl method using Foss automatic distillation equipment), crude fiber (gravimetric), and total carbohydrates (volumetric using Luff Schoorl reagent). The analyses of maize grain composition, using the above mentioned methods, were performed by the “Service of Agricultural Analysis” (Burjassot, Spain) of Generalitat Valenciana (the regional government of Valencia), following their own protocols.

### 4.8. Statistics

The statistical differences between the means of all the treatments were performed by analysis of variances (ANOVA) at the 95% confidence level. Before the ANOVA, data requirements of normality and homogeneity of variances were checked according to Levene’s and Shapiro–Wilk tests. When the null ANOVA hypothesis was rejected, post-hoc comparisons were made to establish any possible statistical differences between the different treatments applied by Tukey’s test. The statistical Statgraphics Centurion XV, version 15.2.05 software program (Statpoint Technologies, Inc., Warrenton, VA, USA) was used to perform the analysis.

## 5. Conclusions

The effectiveness of a new polymer-coated CRF was compared to a nonpolymeric fertilizer produced by a similar manufacturing technology (DURAMON^®^), a traditional fertilizer (NSA), and an untreated CONTROL. No significant differences in crop yields were observed between treatments on the microscale experiment. In the field, however, the yield was higher for the CRF fertilizer than for NSA. The most relevant finding of this work was that the polymeric coating allowed reducing N doses by at least 20% (CRF_r2_) with the same efficacy as that achieved with DURAMON^®^ and NSA applied at standard doses. Easier agronomic crop management than with traditional fertilization and the use of less polluting materials mean that applying this new CRF developed by Fertinagro Biotech—or similar formulations—is especially promising for the extensive cropping of maize guaranteeing yields, although further field studies are needed to confirm the data presented here.

## Figures and Tables

**Table 1:** Effects of fertilizer treatments (CRF and their reductions, DURAMON^®^, NSA) on the growth, the effective quantum yield of photosystem II (ΦPSII), leaf greenness, and N content compared to CONTROL, in the microscale experiment (CRF, DURAMON^®^ and NSA: 350 kg ha^−1^; CRF_r1_: 315 kg ha^−1^; CRF_r2_: 280 kg ha^−1^; CRF_r3_: 245 kg ha^−1^; CRF_r4_: 210 kg ha^−1^). Values are means ± SD (data on growth parameters, *n* = 4 plants; data on photosynthetic and nutritional parameters, *n* = 16 plants) at the phenological stage of 8 nodes.

	CRF	CRF_r1_	CRF_r2_	CRF_r3_	CRF_r4_	DURAMON^®^	NSA	CONTROL
Total fresh weight (aerial part) (g)	647.3 ± 96.8 ^bc^	532.1 ± 140.6 ^abc^	486 ± 184 ^ab^	661.5 ± 60.2 ^c^	486.3 ± 68.2 ^ab^	537.8 ± 127.8 ^abc^	561.2 ± 128.3 ^abc^	400.8 ± 41.5 ^a^
Dry weight (aerial part) (%)	22.8 ± 3.3 ^ab^	24.5 ± 3.8 ^ab^	21 ± 3.1 ^a^	24.4 ± 2.4 ^b^	21.6 ± 1.6 ^a^	23.9 ± 1.4 ^ab^	22.6 ± 1.6 ^ab^	24.7 ± 3.7 ^a^
Total length (cm)	167 ± 10.4 ^ab^	157 ± 14.9 ^ab^	152.5 ± 29.4 ^ab^	176.8 ± 9.2 ^c^	159 ± 8.7 ^bc^	152.3 ± 17.4 ^ab^	154.5 ± 16.1 ^abc^	131.8 ± 9.8 ^a^
Primary stem length (cm)	93 ± 6 ^bc^	75.3 ± 16 ^ab^	71 ± 20.8 ^a^	96.8 ± 6.7 ^c^	79.8 ± 11.8 ^abc^	78 ± 17.5 ^abc^	80.8 ± 16.6 ^abc^	68.1 ± 5.9 ^a^
Stem diameter (mm)	33.2 ± 2.8 ^bc^	30 ± 2.3 ^ab^	31.4 ± 4.3 ^abc^	32.7 ± 3 ^abc^	29.6 ± 2.2 ^ab^	32.3 ± 1.9 ^abc^	33.8 ± 2.3 ^c^	29.4 ± 1.2 ^a^
Leaf weight (g)	15.5 ± 1.6 ^ab^	15.6 ± 2.5 ^ab^	15 ± 2.5 ^ab^	18.8 ± 2.4 ^c^	15.8 ± 1.8 ^ab^	16.2 ± 1 ^bc^	16.6 ± 0.9 ^bc^	13.4 ± 1.1 ^a^
Leaf RWC (%)	77.1 ± 4 ^ab^	76.2 ± 6.2 ^ab^	74.6 ± 5.2 ^ab^	73.1 ± 4.1 ^a^	77 ± 3.9 ^ab^	80.7 ± 8.9 ^b^	78.1 ± 4.1 ^ab^	74.8 ± 2.4 ^ab^
Total foliar area (cm^2^)	29.2 ± 4.3 ^c^	21.2 ± 5.3 ^ab^	22.3 ± 5.2 ^ab^	26.9 ± 3.3 ^bc^	21.3 ± 4.6 ^ab^	25 ± 5.8 ^abc^	25.8 ± 5.1 ^abc^	19.4 ± 2.8 ^a^
ΦPSII	0.67 ± 0.04 ^ab^	0.65 ± 0.07 ^ab^	0.64 ± 0.03 ^a^	0.66 ± 0.04 ^ab^	0.69 ± 0.03 ^b^	0.65 ± 0.08 ^ab^	0.65 ± 0.06 ^a^	0.64 ± 0.07 ^ab^
Leaf greenness (SPAD units)	54.17 ± 4.99 ^a^	51.68 ± 5.24 ^a^	53.91 ± 5.24 ^a^	51.16 ± 5.51 ^a^	52.71 ± 4.83 ^a^	52.94 ± 4.91 ^a^	53.25 ± 3.3 ^a^	50.1 ± 4.73 ^a^
N (%)	2.51 ± 0.3 ^b^	2.11 ± 0.18 ^a^	2.21 ± 0.08 ^ab^	2.11 ± 0.23 ^a^	2.23 ± 0.19 ^ab^	2.04 ± 0.21 ^a^	2.48 ± 0.37 ^b^	2.13 ± 0.26 ^a^

Different letters in the same row indicate significant statistical differences (Tukey’s test, *p* < 0.05).

**Table 2:** Hormone activity comparison between fertilizer treatments applied (CRF, CRF_r2_, NSA) compared to CONTROL. Hormones quantified were indoleacetic acid (*IAA*), jasmonic acid (*JA*), salicylic acid (*SA*), abscisic acid (*ABA*), and cytokinins—including isopentenyl adenine (*iP*), t-zeatin (*tZ*) and dihidrozeatin (*DHZ*). Also, at field level gibberellins (*GA_1_*, *GA_3_*) were quantified (Microscale- CRF, DURAMON^®^ and NSA: 350 kg ha^−1^; CRF_r2_: 280 kg ha^−1^. Field- CRF, NSA: 300 kg ha^−1^; CRF_r2_: 240 kg ha^−1^). Values are means ± SD (*n* leaf pools per treatment = 4) at the end of the culture and expressed in ng hormone g^−1^ FW leaves.

	Microscale				
	CRF	CRF_r2_	DURAMON^®^	NSA	CONTROL
*IAA*	3.52 ± 0.11 ^b^	3.08 ± 0.36 ^b^	3.24 ± 0.65 ^b^	1.85 ± 0.24 ^a^	2.19 ± 0.3 ^a^
*JA*	0.76 ± 0.07 ^a^	0.54 ± 0.08 ^a^	1.67 ± 0.25 ^b^	0.65 ± 0.15 ^a^	0.48 ± 0.06 ^a^
*SA*	39.8 ± 9.68 ^b^	35.87 ± 3.32 ^ab^	39.32 ± 6.58 ^b^	45.48 ± 13.37 ^b^	24.3 ± 5.67 ^a^
*ABA*	10 ± 0.84 ^b^	8.14 ± 0.64 ^ab^	11.62 ± 1.21 ^b^	10.04 ± 0.42 ^ab^	6.61 ± 0.79 ^a^
*iP*	0.26 ± 0.09 ^bc^	0.07 ± 0.02 ^a^	0.19 ± 0.1 ^bc^	0.18 ± 0.05 ^b^	0.29 ± 0.01 ^c^
*tZ*	1.08 ± 0.28 ^b^	0.23 ± 0.08 ^a^	0.45 ± 0.17 ^a^	1.61 ± 0.64 ^c^	0.18 ± 0.06 ^a^
	**Field**				
	**CRF**	**CRF_r2_**		**NSA**	**CONTROL**
*IAA*	1.93 ± 0.14 ^a^	1.83 ± 0.06 ^a^		2.01 ± 0.12 ^a^	2.05 ± 0.24 ^a^
*JA*	3.29 ± 1.7 ^b^	0.76 ± 0.29 ^a^		0.80 ± 0.15 ^a^	2.96 ± 1.09 ^b^
*SA*	142.79 ± 58.74 ^a^	142.45 ± 60.12 ^a^		105.79 ± 14.65 ^a^	159.12 ± 17.61 ^a^
*ABA*	6.07 ± 0.67 ^b^	7.03 ± 0.37 ^c^		6.33 ± 0.76 ^bc^	3.78 ± 0.35 ^a^
*iP*	1.45 ± 0.35 ^b^	0.97 ± 0.22 ^a^		0.92 ± 0.17 ^a^	0.67 ± 0.05 ^a^
*tZ*	3.3 ± 0.4 ^bc^	2.72 ± 0.65 ^b^		3.91 ± 0.81 ^c^	1.19 ± 0.65 ^a^
*DHZ*	0.13 ± 0.02 ^c^	0.08 ± 0.02 ^b^		0.07 ± 0.01 ^b^	0.04 ± 0.01 ^a^
*GA_1_*	3.76 ± 1.54 ^ab^	2.64 ± 1.15 ^ab^		8.51 ± 9.7 ^b^	0.75 ± 0.46 ^a^
*GA_3_*	0.006 ± 0.004 ^a^	0.005 ± 0.001 ^a^		0.019 ± 0.006 ^b^	0.006 ± 0.002 ^a^

Different letters in the same row indicate significant statistical differences (Tukey’s test, *p* < 0.05). DHZ: not detected at the microscale.

**Table 3:** Comparison of growth parameters between fertilizer treatments applied (CRF and their reductions, DURAMON^®^, NSA) compared to CONTROL in the microscale experiment (CRF, DURAMON^®^ and NSA: 350 kg ha^−1^; CRF_r1_: 315 kg ha^−1^; CRF_r2_: 280 kg ha^−1^; CRF_r3_: 245 kg ha^−1^; CRF_r4_: 210 kg ha^−1^). Values are means ± SD (*n* = 12) at the end of the culture.

	CRF	CRF_r1_	CRF_r2_	CRF_r3_	CRF_r4_	DURAMON^®^	NSA	CONTROL
Total fresh weight (aerial part ^1^) (g)	425 ± 44.7 ^abc^	437.8 ± 105 ^abc^	430.7 ± 71 ^abc^	351.2 ± 40 ^ab^	467 ± 22.9 ^c^	384.8 ± 49.4 ^abc^	420.5 ± 71.8 ^abc^	333 ± 65.8 ^a^
Dry weight (aerial part ^1^) (%)	42.4 ± 2.4 ^abc^	43.2 ± 3.4 ^abc^	41.4 ± 0.6 ^a^	44.6 ± 2.7 ^ab^	42.7 ± 1.8 ^abc^	44.7 ± 3 ^c^	41.6 ± 1.2 ^ab^	43.4 ± 1.1 ^abc^
Primary stem length ^2^ (cm)	170.1 ± 5.9 ^a^	178.9 ± 6.1 ^a^	165.1 ± 23.9 ^a^	168.3 ± 11.1 ^a^	182.4 ± 5.2 ^a^	170.8 ± 8 ^a^	166.1 ± 12.6 ^a^	166.3 ± 12.4 ^a^
Stem diameter (mm)	27.2 ± 1.4 ^bc^	26.1 ± 1.7 ^bc^	27.2 ± 1.1 ^bc^	24.7 ± 1 ^ab^	28.1 ± 1 ^c^	26.2 ± 2.6 ^bc^	26.2 ± 2.9 ^bc^	23 ± 1 ^a^
Ear weight ^3^ (g)	213.6 ± 38.3 ^cde^	190 ± 31.2 ^bcd^	225.2 ± 30.3 ^e^	158.3 ± 20 ^ab^	180.9 ± 37.3 ^bcde^	175.9 ± 26 ^bc^	217.4 ± 20.5 ^de^	130.9 ± 14.7 ^a^
Ear length (cm)	20.4 ± 2.3 ^abc^	20 ± 0.9 ^abc^	21.5 ± 1 ^c^	18.7 ± 1 ^ab^	20.1 ± 0.7 ^abc^	18.9 ± 1.5 ^ab^	20.5 ± 1.3 ^bc^	18.6 ± 1.1 ^a^
Total dry grain weight per plant (g)	132.3 ± 27.5 ^c^	120.2 ± 16.7 ^bc^	142.1 ± 24.6 ^c^	90.7 ± 24.1 ^ab^	133.1 ± 27.6 ^c^	114 ± 16.7 ^bc^	144.8 ± 14.2 ^c^	79.2 ± 17.8 ^a^
Weight of 100 grains (g)	27.4 ± 1.9 ^a^	25.7 ± 2.1 ^a^	28.5 ± 1.1 ^a^	27.3 ± 1.6 ^a^	28.5 ± 1.8 ^a^	26.5 ± 2.4 ^a^	27.2 ± 1.4 ^a^	27.3 ± 2.3 ^a^
Grain number per plant × 0.01	4.8 ± 1 ^c^	4.1 ± 0.6 ^c^	4.9 ± 0.8 ^c^	3.1 ± 0.8 ^ab^	4.3 ± 0.7 ^c^	4.3 ± 0.6 ^bc^	5.3 ± 0.5 ^c^	2.9 ± 0.6 ^a^

Different letters in the same row indicate significant statistical differences (Tukey’s test, *p* < 0.05). ^1^ Total tillers without including ears. ^2^ Stem length without including inflorescence. ^3^ Including brackets at the moment of recollection.

**Table 4:** Field harvest comparison of growth parameters and grain yield of *Zea mays* var. *pioneer p0725 short cycle 450* between different fertilizer treatments applied CRF, CRF_r2_ and NSA compared to CONTROL (CRF, NSA: 300 kg ha^−1^; CRF_r2_: 240 kg ha^−1^). Values on means ± SD (*n* = 80) at the end of the culture—90 days after applying the fertilizers.

	CRF	CRF_r2_	NSA	CONTROL
Ear length (cm)	19.21 ± 2.5 ^c^	17.9 ± 1.87 ^ab^	18.6 ± 1.91 ^bc^	17.25 ± 2.34 ^a^
Ear fresh weight (t ha^−1^)	25.48 ± 8.19 ^b^	22.21 ± 6.11 ^a^	22.16 ± 4.93 ^a^	18.88 ± 6.01 ^a^
Ear dry weight (t ha^−1^)	18.4 ± 6.59 ^c^	15.57 ± 4.5 ^b^	14.39 ± 3.57 ^ab^	13.68 ± 5.07 ^a^
Grain dry weight (t ha^−1^)	13.83 ± 5.71 ^c^	11.63 ± 4.12 ^b^	11.41 ± 2.99 ^b^	9.72 ± 4.50 ^a^
Nitrogen Use Efficiency (kg kg^−1^ N)	46.12 ± 19.05 ^a^	49.82 ± 19.66 ^a^	39.53 ± 10.99 ^b^	-

Different letters in the same row indicate significant statistical differences (Tukey’s test, *p* < 0.05).

**Table 5:** Characteristics of the soil used in the experimental analysis from the first 15 cm of soil surface. Data on total nitrogen, total carbon and organic carbon, pH, electrical conductivity (EC), and other macro and micronutrients are shown. Values are means ± SD (*n* = 5) at the beginning of the experiment.

Parameters	Mean ± SD (%)	
	Microscale	Field
Total nitrogen (g kg^−1^)	0.80 ± 0.20	0.50 ± 0.10
Total carbon (g kg^−1^)	18.50 ± 1.20	13.30 ± 0.90
Organic carbon (g kg^−1^)	4.80 ± 0.20	5.60 ± 0.10
pH	8.88 ± 0.04	8.76 ± 0.15
EC (dS m^−1^)	0.14± 0.02	0.15 ± 0.02
P (g kg^−1^)	0.70 ± 0.10	0.50 ± 0.01
K (g kg^−1^)	6.60 ± 0.20	6.00 ± 0.10
Mg (g kg^−1^)	2.47 ± 0.10	1.80 ± 0.03
Ca (g kg^−1^)	35.60 ± 5.40	20.70 ± 4.70
Fe (g kg^−1^)	9.90 ± 0.20	9.90 ± 0.50
Cu (mg kg^−1^)	13.49 ± 0.56	8.65 ± 0.40
Mn (mg kg^−1^)	180.86 ± 4.33	126.52 ± 4.59
Zn (mg kg^−1^)	24.02 ± 0.74	21.82 ± 0.45

## Data Availability

All relevant data are included in the article.
